# Lateralized glymphatic system impairment in acute ischemic stroke: a DTI–ALPS study of upper limb motor function

**DOI:** 10.3389/fneur.2026.1863013

**Published:** 2026-05-28

**Authors:** Zilong Zhu, Zhilong Zhao, Lihua Zhou, Junfeng Xiong, Tianrui Zhang, Chunling Wei, Jianbin Zhang

**Affiliations:** 1 The Second Affiliated Hospital of Nanjing University of Chinese Medicine, Nanjing, Jiangsu, China; 2 Nanjing University of Chinese Medicine, Nanjing, Jiangsu, China

**Keywords:** acute ischemic stroke, DTI-ALPS, glymphatic system, motor function, neuroimaging

## Abstract

**Background:**

The glymphatic system (GS) is a brain-specific waste clearance pathway essential for maintaining cerebral homeostasis. Diffusion tensor imaging analysis along the perivascular space (DTI-ALPS) provides a non-invasive means of evaluating GS function. However, the characteristics of GS impairment in acute ischemic stroke (AIS) and their relationship with clinical function remain incompletely understood.

**Methods:**

Sixty-nine patients with AIS (35 left-sided infarction, 34 right-sided infarction) and 34 healthy controls (HCs) were enrolled. All patients underwent 3.0 T MRI within 72 h of onset. Bilateral DTI-ALPS indices were calculated using an automated ROI algorithm. Neurological and motor functions were assessed with the National Institutes of Health Stroke Scale (NIHSS) and the Fugl-Meyer Assessment-Upper Extremity (FMA-UE). Partial correlation analyses, controlling for age, sex and years of education, examined associations between DTI-ALPS indices and clinical scale scores.

**Results:**

Among right-handed HCs, the left-hemisphere DTI-ALPS index was significantly higher than the right (*P* < 0.0001), indicating physiological lateralization of GS clearance capacity. Compared with HCs, both the left- and right-sided infarction groups exhibited significantly reduced left hemisphere DTI-ALPS indices (*P* < 0.05), whereas right hemisphere indices did not differ significantly regardless of infarct side. In the left infarction group, the left hemisphere DTI-ALPS index was negatively correlated with NIHSS scores (*r* = −0.418, *P* = 0.017) and positively correlated with FMA-UE scores (*r* = 0.392, *P* = 0.026). In the right infarction group, the left hemisphere DTI-ALPS index showed a positive correlation with FMA-UE scores (*r* = 0.513, *P* = 0.003).

**Conclusions:**

These findings suggest physiological lateralization of GS function with left hemispheric predominance in right-handed individuals. AIS produces a left-dominant pattern of GS impairment that is independent of infarct laterality. The left hemisphere DTI-ALPS index may represent a promising, lateralization-independent biomarker for upper limb motor function assessment in the acute phase of stroke.

## 1 Introduction

Acute ischemic stroke (AIS) triggers a cascade of pathological reactions, including inflammatory responses and cytotoxic edema, leading to neuronal damage and neurological deficits (1). During brain tissue injury and repair, metabolic waste products accumulate, and their abnormal buildup has been identified as a major contributor to the development and progression of various neurological disorders (2, 3). Therefore, timely clearance of accumulated metabolic waste and toxic proteins is essential for maintaining white matter tract integrity and promoting neural repair and functional compensation (4, 5). Historically, our understanding of how the central nervous system clears metabolic waste has remained limited. Protected by the blood-brain barrier and lacking typical lymphatic vessels, the brain parenchyma cannot rely on the conventional lymphatic system for waste excretion. Consequently, the brain-specific waste clearance system has become a research focus.

In 2012, Iliff et al. (6) first described the glymphatic system (GS), a brain-specific waste clearance network driven by cerebrospinal fluid (CSF) pulsation along perivascular spaces and aquaporin-4-mediated fluid exchange (7). As the central nervous system lacks conventional lymphatic vessels, the GS is essential for maintaining cerebral homeostasis through metabolic waste removal (8). However, prior GS research relied heavily on invasive tracer-based imaging, limiting clinical translation (9).

In 2017, Taoka et al. (10) proposed DTI-ALPS as a non-invasive imaging assessment method. DTI-ALPS quantifies the diffusion characteristics of water molecules along perivascular spaces by calculating the ratio of diffusion diffusivities in projection and association fibers along the X, Y, and Z axes. Because of its noninvasiveness, reproducibility, and absence of contrast agent–allergy risks, this index has become an important biomarker for clinical GS function assessment and disease progression prediction (11). Recent studies have demonstrated that GS dysfunction is closely related to the pathophysiological processes of cerebral infarction (9, 12). Following cerebral infarction, blood-brain barrier disruption, cerebral edema formation, neuroinflammation, and accumulation of neurotoxic factors can impair glymphatic function and induce hemispheric asymmetry (13).

Although previous studies have linked GS dysfunction to ischemic stroke, the hemispheric asymmetry of GS and its association with motor function in AIS remain unclear. Therefore, we utilized DTI-ALPS combined with clinical scales to quantitatively evaluate GS function in patients with left- or right-sided infarction. We aimed to characterize GS impairment patterns, explore their correlations with neurological and motor deficits, and clarify the role of the GS in AIS pathophysiology to support clinical assessment and therapeutic target discovery.

## 2 Materials and methods

### 2.1 Study participants

This cross-sectional, single-center study was conducted from June 2024, with an anticipated completion date of October 2025. It was approved by the Ethics Committee of the Second Affiliated Hospital of Nanjing University of Chinese Medicine (approval No. 2024SEZ-003-01) and adhered to the principles of the Declaration of Helsinki. Informed consent was obtained from all participants. This trial was registered in the Chinese Clinical Trials Registry (registration No. ChiCTR2400085342).

The inclusion criteria for the AIS group were as follows: Diagnosed with acute ischemic stroke according to the Chinese Guidelines for Diagnosis and Treatment of Acute Ischemic Stroke 2023; Aged 40–85 years, either sex, and right-handed (to minimize the confounding effects of handedness on motor function assessment); First-ever ischemic stroke with unilateral lesions in the basal ganglia and/or corona radiata, with clinical evaluation and magnetic resonance imaging (MRI) performed within 72 h of onset; Provision of informed consent following detailed explanation of study objectives, methods, potential risks, and benefits; Normal consciousness, stable vital signs, and ability to cooperate with treatment, functional assessment, and neuroimaging data acquisition.

Exclusion criteria for the AIS group were as follows: Neurological dysfunction caused by brain tumors, cerebral parasitosis, or other diseases confirmed clinically; Severe cardiac, pulmonary, hepatic, or renal insufficiency, or other severe diseases in the acute phase; Cognitive impairment, psychiatric disorders, or other conditions preventing cooperation with treatment, functional assessment, and neuroimaging examination; Presence of cardiac pacemakers, metallic implants, or severe agitation precluding MRI; Poor MRI image quality with significant motion artifacts; Cerebral hemorrhage, transient ischemic attack (TIA), history of stroke, or severe white matter hyperintensity; Concurrent participation in other clinical trials.

The inclusion criteria for the HCs group were: Absence of neurological diseases confirmed by imaging and clinical assessment, and right-handedness; Ability to understand and complete imaging examinations; Voluntary participation in the study with signed informed consent; and age between 40 and 85 years. The exclusion criteria included poor quality of MRI images, claustrophobia, or other MRI contraindications.

### 2.2 Data collection and processing

#### 2.2.1 Clinical assessment

Patients with AIS completed the assessments of the NIHSS and the FMA-UE. All assessments were conducted by two deputy chief physicians with appropriate clinical training and experience.

#### 2.2.2 MRI acquisition

MRI data were acquired using a 3.0-T scanner (MAGNETOM Vida, Siemens) equipped with a 32-channel head coil. Identical protocols were applied to AIS patients and HCs, including: 3D T1-weighted (T1WI), T2-weighted (T2WI), diffusion-weighted (DWI), fluid-attenuated inversion recovery (FLAIR), and diffusion tensor imaging (DTI).

3D T1-weighted imaging data were acquired with a fast spoiled gradient echo sequence: field of view = 250 mm × 250 mm, repetition time = 1,900 ms, echo time = 3.82 ms, slice thickness = 1 mm, no slice spacing, voxel size = 1 × 1 × 1 mm^3^, matrix = 256 × 256.

Diffusion MRI data were acquired with a single-shot echo-planar imaging sequence: field of view = 256 mm × 256 mm, repetition time = 10,000 ms, echo time = 90 ms, slice thickness = 2.0 mm, no gap, number of slices = 74, voxel size = 2 × 2 × 2 mm^3^, matrix = 128 × 128, 30 gradient directions at b = 1,000 s/mm^2^, one b0 image at b = 0 s/mm^2^. A board-certified neuroradiologist performed visual quality control for all datasets before analysis.

#### 2.2.3 MRI data processing

DICOM data were converted to NIfTI format using dcm2niigui. DTI data processing was performed using the FMRIB Software Library (FSL, version 5.0.9, http://www.fmrib.ox.ac.uk/fsl) and MRtrix3. Preprocessing included: data quality inspection, denoising, Gibbs artifact removal, eddy current correction, motion correction, and bias field correction. Brain extraction was performed using the BET tool to remove non-brain tissue. Tensor fitting was conducted using FSL's DTIFIT command to generate fractional anisotropy (FA), mean diffusivity (MD), axial diffusivity (AD), radial diffusivity (RD), and diffusion tensor components along x, y, and z axes (Dxx, Dyy, Dzz), followed by color map creation.

**Figure 1:**
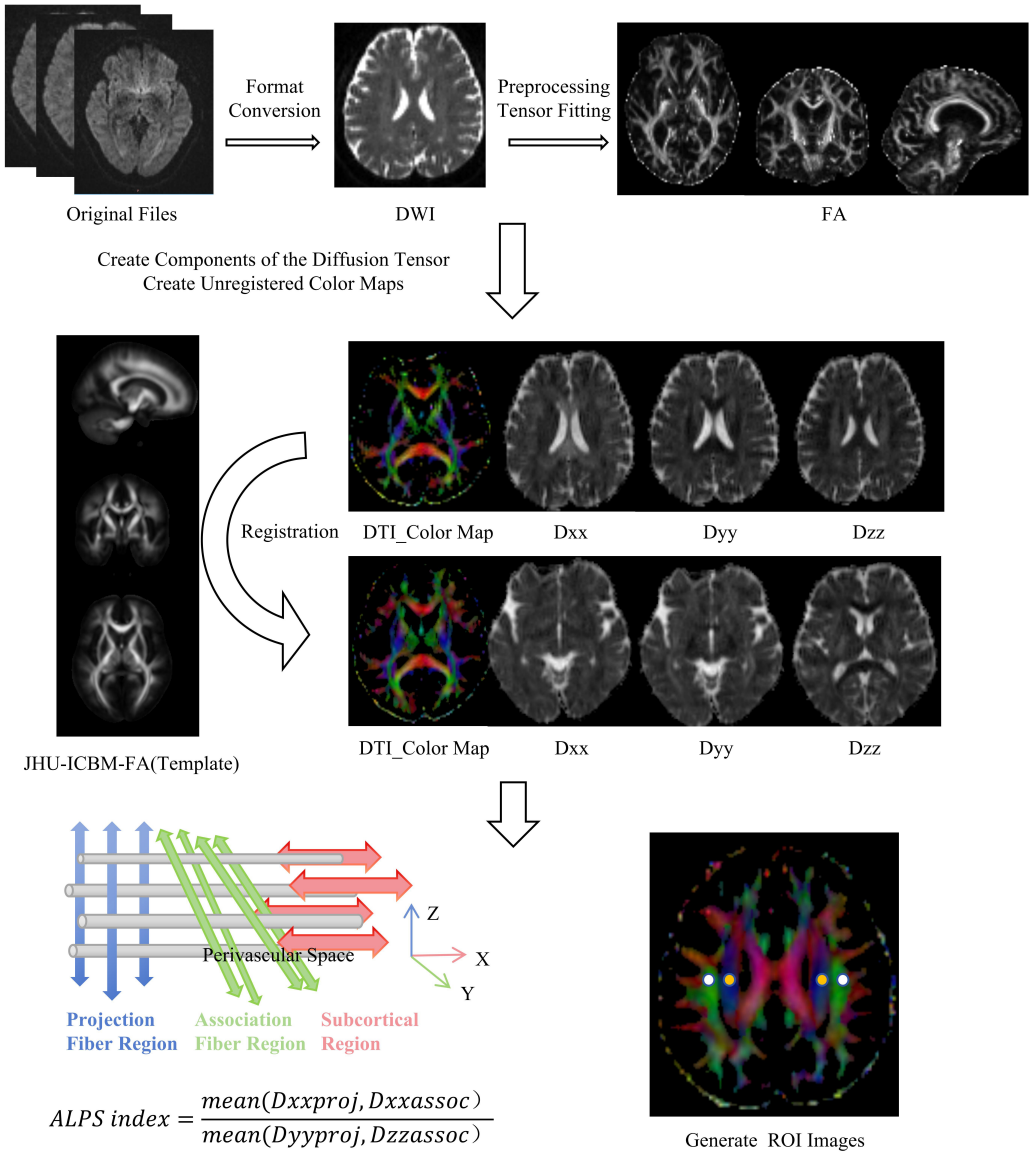
DTI-ALPS data analysis principles and flowchart. DWI, Diffusion weighted imaging; FA, fractional anisotropy; ROI, region-of-interest; JHU, Johns Hopkins University.

FSL's FLIRT tool was used to register FA maps to MNI standard space using the JHU-ICBM-FA-1mm atlas as reference, generating transformation matrices. These matrices were applied to register Dxx, Dyy, and Dzz to Montreal Neurological Institute (MNI) space (14). This study used an automated algorithm developed in MATLAB (MathWorks, Natick, MA, USA) for region-of-interest (ROI) placement to eliminate subjective bias associated with manual delineation. The specific steps were as follows: According to the DTI RGB color map, analysis was strictly confined to a specific Z-axis plane (95–105, corresponding to the body of the lateral ventricle), we defined specific X-Y regions in the left and right cerebral hemispheres. By reading the registered DTI color map, the software automatically identified and output the 3D coordinates with the highest values for projection and association fibers as the final ROIs, and then calculated the ALPS index. The detailed code is provided in Supplementary File 1. DTI-ALPS indices were automatically calculated using the following formula. Detailed procedures and the calculation formula are illustrated in Figure 1.

### 2.3 Statistical analysis

Data were analyzed using SPSS 26.0 and Microsoft Excel 2019. Categorical variables (e.g., sex) were analyzed using chi-square tests. Continuous variables were first tested for normality. Normally distributed data with homogeneity of variance were expressed as mean ± standard deviation (SD) and compared between groups using independent-samples *t*-tests and one-way analysis of variance (ANOVA). Non-normally distributed data were presented as median (interquartile range) and compared using the Mann–Whitney U test or Kruskal–Wallis test. Partial correlation analysis, controlling for age, sex, and education years as covariates, was performed to examine relationships between DTI-ALPS indices and clinical scale scores with significance set at *P* values < 0.05 (two-tailed).

## 3 Results

### 3.1 Clinical and demographic data

This study enrolled 103 participants, including 34 HCs, 35 patients with left hemispheric stroke (LHS), and 34 patients with right hemispheric stroke (RHS). Table 1 summarizes the demographic and clinical characteristics of the participants. No significant differences were found in age, sex, years of education, or comorbidities between-group (*P* > 0.05). No significant differences were observed in NIHSS or FMA-UE scores between the LHS group and the RHS group (*P* > 0.05).

**Table 1:** Participants' characteristics.

Demographics	HCs	LHS	RHS	*P*-value
	(*N* = 34)	(*N* = 35)	(*N* = 34)	
Lesion location		Left	Right	
Age (years)	62.2 ± 9.0	67.0 ± 9.1	63.6 ± 9.7	0.0878
Sex (male/female)	18/16	24/11	27/7	0.0657
Education years	8 (5, 8)	8 (5, 11)	8 (5, 11)	0.4985
Hypertension	14	21	22	0.1180
Diabetes mellitus	6	12	11	0.2463
Coronary heart disease	4	5	1	0.338
Smoking	8	10	12	0.5633
Alcohol intake	6	11	7	0.3603
Thrombolysis/thrombectomy		8	10	0.5353
Stroke severity				0.5548
Mild (1, 2, 3, 4)		23	20	
Moderate (5, 6, 7, 8, 9, 10, 11, 12, 13, 14, 15)		12	14	
FMA-UE		43.37 ± 20.16	41.97 ± 18.45	0.7645
NIHSS		4 (2, 7)	4 (2.75, 5.25)	0.6838

LHS, left hemispheric stroke; RHS, right hemispheric stroke; NIHSS, National Institutes of Health Stroke Scale; FMA-UE, Fugl-Meyer Assessment-Upper Extremity.

### 3.2 Comparison of DTI-ALPS indices among the HCs, LHS, and RHS group

Among 34 right-handed healthy controls, comparison of bilateral DTI-ALPS indices revealed that the left hemisphere DTI-ALPS index was significantly higher than the right (*P* < 0.0001) (Table 2). Comparisons between the AIS group and HCs group showed that both the LHS and RHS groups had significantly reduced left hemisphere DTI-ALPS indices compared with controls (*P* < 0.05). However, right hemisphere DTI-ALPS indices showed no significant differences from HCs group regardless of infarct side (*P* > 0.05) (Table 2).

**Table 2:** Comparison of DTI-ALPS indices among the three groups.

DTI-ALPS index	HCs	LHS	RHS	*P-*value		
	(*N* = 34)	(*N* = 35)	(*N* = 34)	HCs vs. LHS	HCs vs. RHS	LHS vs. RHS
ALPS_L	2.547 ± 0.5997^a^	2.115 ± 0.5477	2.193 ± 0.5867	0.0069^**^	0.035^*^	0.839
ALPS_R	1.318 ± 0.1977	1.299 ± 0.1938	1.392 ± 0.2847	0.3024	0.979	0.2169

LHS, left hemispheric stroke; RHS, right hemispheric stroke; ^a^*P* < 0.0001 for bilateral comparison in the HCs group; ALPS_L, the left ALPS-index; ALPS_R, the right ALPS-index; ^*^P < 0.05; ^**^P < 0.01.

### 3.3 Correlation analysis of DTI-ALPS and clinical features in AIS group

**Figure 2:**
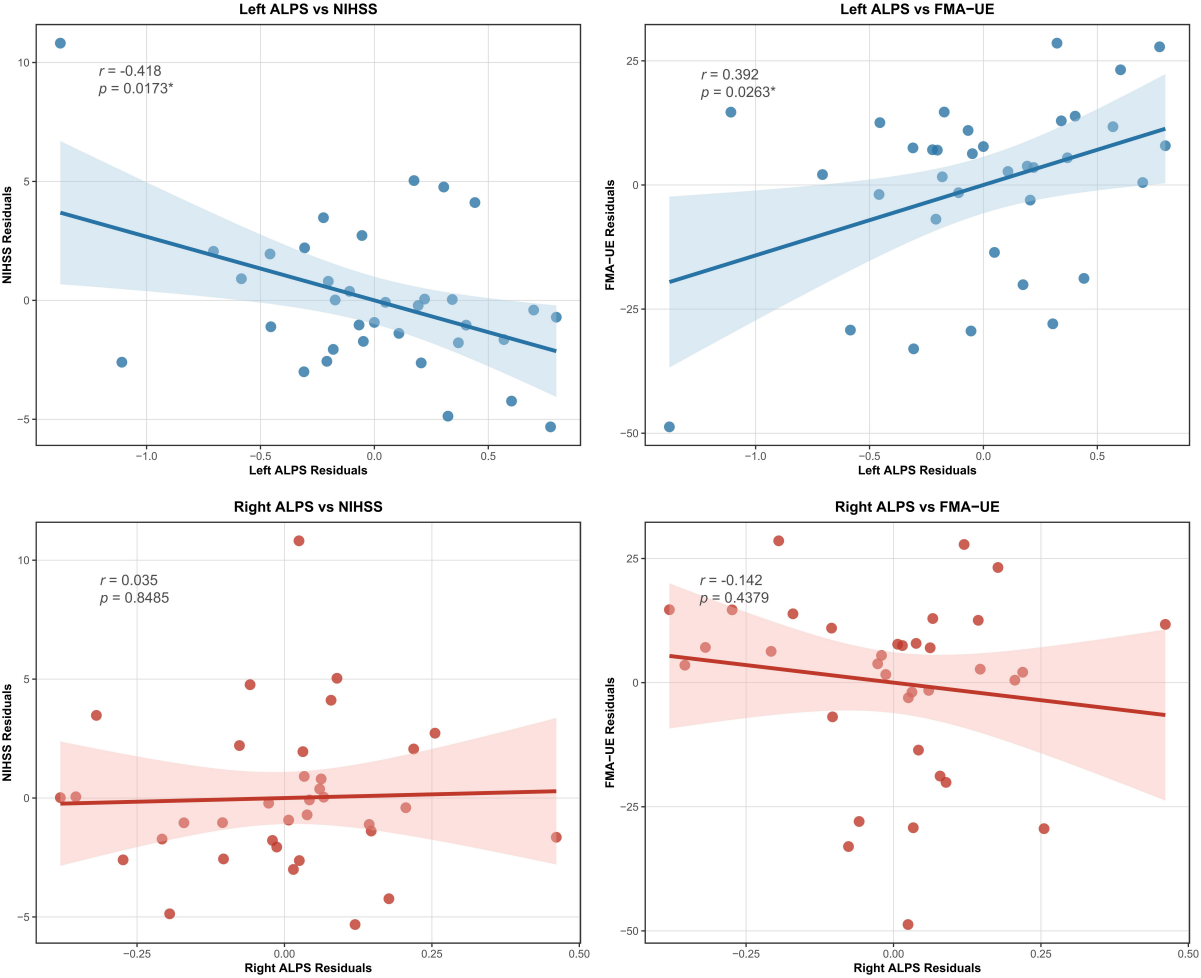
Partial correlation analyses between bilateral DTI-ALPS indices and NIHSS, FMA-UE in patients with left-sided cerebral infarction. NIHSS, National Institute of Health stroke scale; FMA-UE, Fugl-Meyer Assessment Upper Extremity Scale. All analyses were adjusted for confounding variables. Shaded areas represent 95% confidence intervals of the regression fitting line. *P < 0.05.

Partial correlation analysis was conducted in the AIS group, controlling for sex, age, and years of education. In the LHS group, the left hemisphere ALPS index showed a moderate negative correlation with admission NIHSS scores (*r* =-0.418, *P* = 0.0173), whereas the right hemisphere ALPS index showed no significant correlation (*P* > 0.05). The left hemisphere ALPS index showed a significant positive correlation with FMA-UE scores (*r* = 0.392, *P* = 0.0263), whereas the right hemisphere ALPS index showed no significant correlation (*P* > 0.05) (Figure 2).

**Figure 3:**
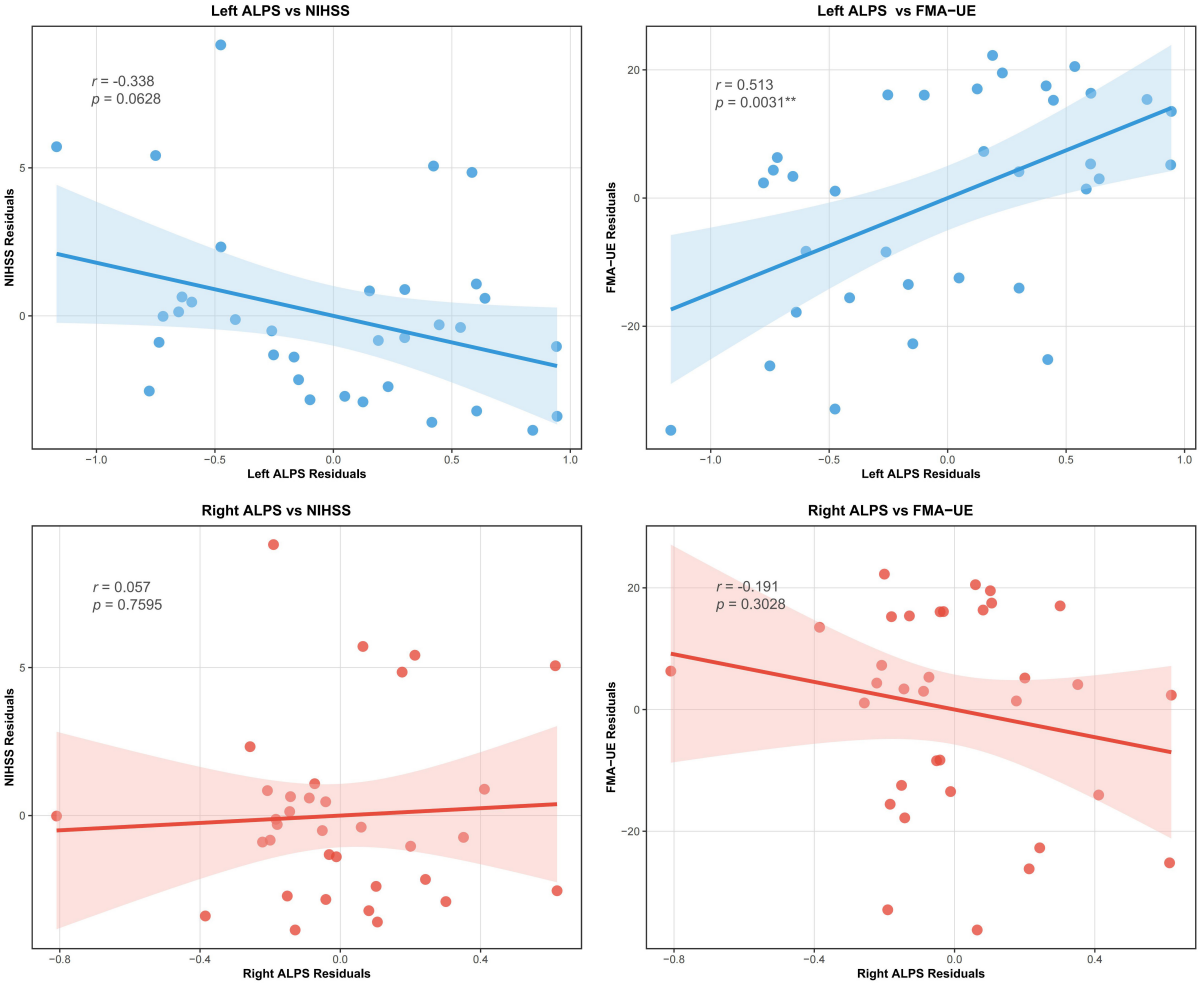
Partial correlation analyses between bilateral DTI-ALPS indices and NIHSS, FMA-UE in patients with right-sided cerebral infarction. NIHSS, National Institute of Health stroke scale; FMA-UE, Fugl-Meyer Assessment Upper Extremity Scale. All correlations were calculated using residual values after adjustment for relevant confounding factors. The shaded bands surrounding the regression lines represent the 95% confidence intervals. *P < 0.05; **P < 0.01.

In the RHS group, DTI-ALPS indices of bilateral hemispheres showed no significant correlation with NIHSS scores (*P* > 0.05). The left hemisphere ALPS index showed a significant positive correlation with FMA-UE scores (*r* = 0.513, *P* = 0.0031), whereas the right hemisphere ALPS index showed no significant correlation (*P* > 0.05) (Figure 3).

## 4 Discussion

The present study evaluated intergroup differences in DTI-ALPS indices to explore hemispheric variations in glymphatic function. The results revealed that the ALPS index in the left cerebral hemisphere of the HCs group was significantly higher than that in the right hemisphere, indicating lateral asymmetry in glymphatic system function in the normal brain. The impact of AIS on the glymphatic system exhibited a left-dominant pattern of impairment rather than a merely localized effect restricted to the ipsilesional hemisphere. The ALPS index in the left cerebral hemisphere correlated with upper limb motor function scores, demonstrating its value as an assessment tool. Furthermore, this correlation was independent of the side of the infarction, offering a new perspective on the pathophysiological mechanisms and therapeutic targets of ischemic stroke.

In the present cohort of right-handed healthy participants, the DTI-ALPS index was significantly higher in the left hemisphere than in the right hemisphere, indicating distinct physiological hemispheric lateralization of glymphatic function. This finding is consistent with previous reports (15). As the central organ of the nervous system, the human brain exhibits prominent functional and structural asymmetry between the two hemispheres (16). In right-handed individuals, the left hemisphere generally serves as the dominant hemisphere. Although genetic predispositions contribute, this lateralization is mainly shaped by long-term task-specific utilization, including repetitive motor tasks, fine movement execution, and language processing, which continuously activate and strengthen left-hemisphere neural circuits (17, 18).

Hand dominance and hemispheric specialization are closely linked and jointly shape motor function (19). Asymmetric interhemispheric connectivity and white matter structure provide the neurobiological basis for functional lateralization (19, 20). The dominant left hemisphere has a more compact and complex white matter architecture (21). Since DTI-ALPS ROIs are placed at fiber-crossing regions, the ALPS index is sensitive to white matter integrity, which partly explains the higher left-hemisphere ALPS index in healthy right-handed individuals (22). In addition, the left hemisphere dominates fine motor and language functions, requiring higher metabolism and blood flow, which further enhances perivascular fluid flow and glymphatic clearance (23–25). Together, these factors underpin the physiological left-lateralized glymphatic pattern observed in this study.

A key observation of this study is that left-hemisphere DTI-ALPS indices were significantly reduced in AIS patients regardless of infarct side, whereas right-hemisphere ALPS indices did not differ significantly from healthy controls. This pattern indicates that acute stroke produces both direct and indirect glymphatic impairment, which can be mechanistically dissociated. Direct local injury following left-hemisphere infarction involves several mechanisms. In acute stroke, cytotoxic edema and neuroinflammation exacerbate structural damage and impair glymphatic clearance (26). In patients with left-hemisphere infarction, the lesion directly damages perilesional and distal white matter tracts, including the corticospinal tract and association fibers, leading to demyelination and fiber disruption, which in turn reduces the ALPS index (27). Thus, left infarction causes direct local injury to left-hemisphere glymphatic function, whereas contralesional effects on the right hemisphere remain mild in the acute phase, consistent with prior observations (28). Indirect transcallosal diaschisis following right-hemisphere infarction represents another key mechanism. Interestingly, right-hemisphere infarction also led to reduced left-hemisphere ALPS indices, whereas the ipsilesional right hemisphere showed no significant difference from healthy controls. This dissociation is best explained by indirect transcallosal mechanisms. All patients were right-handed, and the dominant left hemisphere exhibits a higher metabolic rate and cerebral blood flow under physiological conditions, rendering it more vulnerable to acute pathological stress (23–25). Moreover, the two hemispheres are tightly interconnected via the corpus callosum and other commissural fibers. Right-hemisphere infarction may induce transcallosal diaschisis, whereby acute disruption of interhemispheric inhibitory balance suppresses neuronal activity in the contralateral left hemisphere and triggers global brain network dysregulation (29, 30), thereby indirectly impairing left-hemisphere glymphatic function. Transcallosal diaschisis is a well-established phenomenon in acute stroke, characterized by depressed metabolism and altered connectivity in structurally intact regions remote from the primary lesion (31, 32). In contrast, early right-hemisphere lesions remain relatively localized, and secondary injury may not have peaked, explaining the preserved right ALPS index. Previous studies indicate that cerebral edema develops within 24–48 h after ischemia and peaks at 72–96 h, accompanied by severe glymphatic impairment (33, 34) Other research has reported reduced right-hemisphere ALPS indices in patients with subacute right-hemisphere infarction (35). Since our study enrolled only acute patients within 72 h, secondary structural and functional damage may not have reached maximal severity, resulting in non-significant early changes in the right hemisphere.

In patients with left-hemisphere infarction, the left ALPS index was significantly negatively correlated with NIHSS scores. In the RHS group, the left ALPS showed no significant correlation with NIHSS scores. These findings support the value of left-hemisphere ALPS in evaluating acute neurological deficits in the context of hemispheric lateralization. Notably, left ALPS index was positively correlated with FMA-UE scores regardless of infarct side, indicating robust and reliable association. These results indicate that left-hemisphere glymphatic function is closely linked to upper limb motor performance and that the left ALPS index serves as an infarct-side-independent biomarker. Previous studies in subacute stroke also reported positive correlations between left ALPS and FMA-UE scores (28). Another study of 59 subacute stroke patients (27 left, 32 right) found that the ipsilesional ALPS index correlated positively with FMA-UE (13). The ALPS index reflects microstructural integrity in periventricular white matter regions that overlap with key segments of the corticospinal tract (10). The structural integrity of the corticospinal tract is a well-established neuroimaging marker for assessing and predicting upper limb motor recovery after stroke (36–38). Therefore, the left ALPS index integrates both glymphatic function and white matter tract integrity, making it particularly informative for motor evaluation.

## 5 Limitations

This study has several limitations. First, the sample size was relatively small, and no stratification analysis was performed for confounding factors such as infarct volume and etiological classification; therefore, the lack of a significant trend observed in the ALPS index for the right hemisphere may reflect insufficient statistical power rather than a true pattern of the effect. Second, we measured ALPS indices only within 72 h of onset, which precludes characterization of longitudinal glymphatic dynamics. Some clinically meaningful trends did not reach statistical significance, likely because of the small sample size and a single early time point. Third, the DTI-ALPS index reflects regional glymphatic function and provides limited information about global brain glymphatic network characteristics. Future studies with larger multicenter samples, longitudinal follow-up, quantitative infarct volumetry, and combined molecular or metabolic markers are warranted to validate these findings and clarify the dynamic role of the glymphatic system in post**-**stroke neurological recovery.

## 6 Conclusions

In conclusion, our findings suggest that the GS clearance capacity in the left-dominant hemisphere exhibits physiological lateralization. Regardless of left or right hemisphere infarction, patients in the acute phase (within 72 h) showed significantly reduced ALPS indices in the left hemisphere compared with healthy controls, whereas no significant differences were observed in right-hemisphere indices. Furthermore, the left-hemisphere ALPS index may represent a promising, lateralization-independent biomarker for assessing upper limb motor function.

## Data Availability

The original contributions presented in the study are included in the article/Supplementary material, further inquiries can be directed to the corresponding authors.

## Supplementary material

Supplementary file 1 Detailed code in matlab.
