# Supplementary material for: Lateralized glymphatic system impairment in acute ischemic stroke: a DTI–ALPS study of upper limb motor function
Source: Front Neurol. 2026 May 28;17:1863013. doi: 10.3389/fneur.2026.1863013 (PMC13253242; doi:10.3389/fneur.2026.1863013)
Supplement: Supplementary file 1 — Detailed code in matlab. [file Table_1.DOCX]

clear; clc;

%%

% %% 主程序部分

root_path = 'DShareFDTI_ALPSdata'; % 根路径

keyword = 'sub'; % 查找包含的关键字

%% 以下不要修改

z_list = 95105; % z轴范围

% 遍历文件夹，寻找包含关键字的子目录

dirs = dir(root_path);

dirs = dirs([dirs.isdir]); % 只获取文件夹

for i = 1length(dirs)

dir_name = dirs(i).name;

if contains(dir_name, keyword)

disp(['处理目录 ', dir_name]);

l_max_mean = 0; % 初始化左侧最大均值

r_max_mean = 0; % 初始化右侧最大均值

path_colormap = fullfile(root_path, dir_name, 'DTI_reoriented_ColorMap.nii.gz');

% 检查文件是否存在

if exist(path_colormap, 'file') ~= 2

disp(['文件不存在 ', path_colormap]);

continue; % 如果文件不存在，跳过该目录

end

% 查找左侧最大均值位置

for z = z_list

data = load_and_pad(path_colormap);

if isempty(data)

continue;

end

[a_l_position, p_l_position, l_max_meangb] = find_max_mean(data(, , z), 120135, 100125, 100120);

if l_max_meangb l_max_mean

l_max_mean = l_max_meangb;

a_l_max_position = [a_l_position, z];

p_l_max_position = [p_l_position, z];

end

end

% 确保位置变量被赋值

if isempty(a_l_max_position) isempty(p_l_max_position)

disp('未找到有效的位置');

continue;

end

% 查找右侧最大均值位置

for z = z_list

data = load_and_pad(path_colormap);

if isempty(data)

continue;

end

[a_r_position, p_r_position, r_max_meangb] = find_max_mean(data(, , z), 4660, 5769, 100120);

if r_max_meangb r_max_mean

r_max_mean = r_max_meangb;

a_r_max_position = [a_r_position, z];

p_r_max_position = [p_r_position, z];

end

end

% 绘制左侧和右侧的ROI图像并保存

plot_roi(path_colormap, [root_path, dir_name, '_l.png'], a_l_max_position(1), p_l_max_position(1), a_l_max_position(2), a_l_max_position(3));

plot_roi(path_colormap, [root_path, dir_name, '_r.png'], a_r_max_position(1), p_r_max_position(1), a_r_max_position(2), a_r_max_position(3));

% 计算扩散张量的相关值

path_Dxx = fullfile(root_path, dir_name, 'dti_reoriented_Dxx.nii.gz');

path_Dyy = fullfile(root_path, dir_name, 'dti_reoriented_Dyy.nii.gz');

path_Dzz = fullfile(root_path, dir_name, 'dti_reoriented_Dzz.nii.gz');

dxx_l_a = calculate_circle_mean(a_l_max_position, path_Dxx);

dxx_l_p = calculate_circle_mean(p_l_max_position, path_Dxx);

dyy_l_p = calculate_circle_mean(p_l_max_position, path_Dyy);

dzz_l_p = calculate_circle_mean(a_l_max_position, path_Dzz);

alps_l = (dxx_l_a + dxx_l_p) (dyy_l_p + dzz_l_p);

dxx_r_a = calculate_circle_mean(a_r_max_position, path_Dxx);

dxx_r_p = calculate_circle_mean(p_r_max_position, path_Dxx);

dyy_r_p = calculate_circle_mean(p_r_max_position, path_Dyy);

dzz_r_p = calculate_circle_mean(a_r_max_position, path_Dzz);

alps_r = (dxx_r_a + dxx_r_p) (dyy_r_p + dzz_r_p);

% 输出计算结果

disp([dir_name,' 左侧ALPS值 ', num2str(alps_l)]);

disp([dir_name,' 右侧ALPS值 ', num2str(alps_r)]);

% Create a table to hold your results

result_table = table();

% Define the data to be written to the CSV (adjust based on your data structure)

result_table.Name = {dir_name}; % Save the directory name (e.g., HC0001)

result_table.Left_Position = {a_l_max_position}; % Assuming this is a numeric array

result_table.Right_Position = {a_r_max_position}; % Assuming this is a numeric array

result_table.Dxx_Left = dxx_l_a;

result_table.Dxx_Right = dxx_r_a;

result_table.Alps_Left = alps_l;

result_table.Alps_Right = alps_r;

% Specify the path to save the CSV

result_file = fullfile(root_path, 'results.csv');

% Check if the CSV file exists, if not, create it with a header

if exist(result_file, 'file') == 2

% Append to existing file

writetable(result_table, result_file, 'WriteRowNames', false, 'WriteMode', 'append');

else

% Write to a new file with headers

writetable(result_table, result_file, 'WriteRowNames', false);

end

end

end
